# Onset Time of Lumbar Erector Spinae Plane Block Compared with Its Thoracic Counterpart: Case Reports

**DOI:** 10.3390/healthcare11081158

**Published:** 2023-04-18

**Authors:** Wei-Chen Chung, Yi-Jie Kuo, Shun-Ming Chan, Jin-De Hou, Ting-Hsun Lin, Jui-An Lin

**Affiliations:** 1 Department of Anesthesiology, Wan Fang Hospital, Taipei Medical University, Taipei 116, Taiwan; 2 Department of Orthopedic Surgery, Wan Fang Hospital, Taipei Medical University, Taipei 116, Taiwan; 3 Department of Orthopedic Surgery, School of Medicine, College of Medicine, Taipei Medical University, Taipei 110, Taiwan; 4 Department of Anesthesiology, Tri-Service General Hospital and National Defense Medical Center, Taipei 11490, Taiwan; 5 Division of Anesthesiology, Hualien Armed Forces General Hospital, Hualien 97144, Taiwan; 6 Department of Anesthesiology, School of Medicine, National Defense Medical Center, Taipei 11490, Taiwan; 7 Department of Anesthesiology, Chung Shan Medical University Hospital, Taichung 40201, Taiwan; 8 Department of Anesthesiology, School of Medicine, Chung Shan Medical University, Taichung 40201, Taiwan; 9 Center for Regional Anesthesia and Pain Medicine, Chung Shan Medical University Hospital, Taichung 40201, Taiwan; 10 Department of Anesthesiology, School of Medicine, College of Medicine, Taipei Medical University, Taipei 110, Taiwan; 11 Center for Regional Anesthesia and Pain Medicine, Wan Fang Hospital, Taipei Medical University, Taipei 116, Taiwan

**Keywords:** lumbar vertebrae, nerve block: erector spinae plane block, nerve block: onset time

## Abstract

The erector spinae plane block (ESPB) at the level of the fifth thoracic vertebra (T5) is a novel technique, first published in 2016, which was found to be effective in both acute and chronic pain control. The mechanism of action and spread of local anesthetic of the ESPB at the lumbar region are thought to differ from those of the thoracic ESPB; however, the difference in onset time has never been evaluated. As for the onset of lumbar ESPBs, we presented three cases: two received lumbar ESPBs (one with chronic low back pain and one with acute postoperative hip pain), and the third one with chronic back pain received a thoracic ESPB. We administered 30 mL of 0.3% ropivacaine in all three patients, but the analgesic effect did not reach its maximum until 3 and 1.5 h, respectively, in the lumbar ESPB cases. On the contrary, the thoracic ESPB case experienced noticeable pain relief within 30 min. The onset time was considerably longer than that reported in earlier reports on ESPBs, and the lumbar ESPB achieved its peak effect much later than the thoracic ESPB using the same formula of local anesthetic. While the delayed-onset lumbar ESPB may have some drawbacks for treating acute postoperative pain, it still could produce significant analgesia, once it took effect, when given to patients suffering from hip surgery with large incisions and intractable low back pain. The current data suggested that the onset time of a lumbar ESPB may be delayed compared with its thoracic counterpart. Therefore, the local anesthetic formula and injection timing should be adjusted for a lumbar ESPB when applied in the perioperative period to make the onset of the analgesic effect coincide with the immediate postoperative pain. Without this concept in mind, clinicians may consider a lumbar ESPB to be ineffective before it takes effect, and consequently treat the patients inadequately with this technique. Future randomized controlled trials should be designed according to our observations to compare lumbar ESPB with its thoracic counterpart regarding onset time.

## 1. Introduction

Pain management is a complex challenge in clinical care. Acute pain is an unpleasant sensation resulting from tissue trauma or inflammation. If not adequately treated, it may lead to a chronic pain condition [1]. According to the review by Cohen S.P. et al., the prevalence of chronic pain varies from 11% to 40% between different countries and references [1]. Intractable chronic pain interferes with patients’ ability to work and their life quality. For instance, failed back surgery syndrome may be caused by multiple etiological factors; therefore, it remains challenging to provide effective treatment [2,3]. Due to the limitations in management options for intractable chronic pain, analgesic techniques are constantly being developed and refined. 

The erector spinae plane block (ESPB) at the fifth thoracic vertebra (T5) level is a novel technique that was first described by Forero M. et al. in 2016 [4]. Local anesthetics were injected at the facial plane of transverse processes, and would spread in a cephalocaudal direction along the plane. Patients with severe pain could reach noticeable pain relief in less than 30 min in that study. After the first report on the thoracic level, the ESPB has been widely used in different pain control situations, including for acute [5] and chronic [6] pain. The result from a meta-analysis concluded that ESPBs significantly lowered the acute postoperative pain score and postoperative nausea and vomiting in different surgeries compared with the control group [5]. Viderman D. reviewed 43 cases receiving ESPBs for chronic pain relief [6]. Although more precise and well-designed studies were needed to make a firm conclusion, the ESPB seemed to be effective for multiple chronic pain conditions. Not long after the original publication, the technique was also found to be effective at the lower thoracic [7,8] and lumbar levels [9,10,11,12]. In more recent years, the possible advantages of lumbar ESPBs in patients receiving lumbar spine surgery [13,14,15] or hip surgery [16] were reported by many studies. However, researchers focused primarily on lumbar ESPBs’ analgesic efficacy, and there was little discussion on the clinical differences between thoracic and lumbar ESPBs. Kose et al. reviewed the differences in the mechanism of action between thoracic and lumbar ESPBs, and reported poorer visualization of the spread of the local anesthetic agent, and varying effects achieved after lumbar ESPB [17].

As of yet, the variation in the onset of action for thoracic and lumbar ESPBs has not been thoroughly described in clinical situations. The optimal concentration and volume of local anesthetic required for lumbar ESPBs have also not been determined. We experienced delayed onset of lumbar ESPBs in both chronic and acute pain scenarios. This report demonstrates two cases of lumbar ESPBs, one with chronic pain and the other with acute pain, where the delayed onset of action was observed compared with that of the thoracic ESPB. The comparison of cases was facilitated by selecting cases with the same injection formula. 

## 2. Methods 

### 2.1. Case Recruitment and Pain Measurement 

We selected cases that were treated with the same local anesthetic formula, in order to facilitate a comparison among patients in different settings. Those with dementia or active psychosis were excluded to avoid reporting bias. Three cases were selected for investigation. We compared cases 1 and 3 based on their similar chronic pain conditions (both suffered from failed back surgery syndrome) and older age. A difference in the onset time between the lumbar and thoracic ESPB was found, and we further surveyed case 2, who was younger, and in a post-surgical acute pain situation. Interestingly, a similar delayed effect of the lumbar ESPB was noted. In all cases, before and after the ESPB, the pain score on the same region was rated on a numeric rating scale. All of the injections were performed under standard vital sign monitoring. The TMU-Joint Institutional Review Board approved the report of this study for all three cases (TMU-JIRB No.: N201808001).

### 2.2. Technical Description of ESPB

This series of ESPBs was all performed by the same anesthesiologist, who had expertise in ultrasound-guided nerve blocks. With patients lying in the prone position, the ultrasound transducer, microconvex-array for thoracic ESPB, as well as curved-array for lumbar ESPB, were used to identify the transverse process of the index level. The ESPB was performed by inserting a 22-gauge spinal needle in-plane toward the transverse process tip, with a transducer in the longitudinal orientation for the lumbar ESPB and transverse orientation for the thoracic ESPB. While the tip of the needle touched the transverse process, 1–2 mL of 5% dextrose water was injected as the test solution, and 30 mL of 0.3% ropivacaine (with 1:400,000 epinephrine) was injected in all three cases after visualizing a linear test spread deep to the erector spinae muscle, separating it from the transverse process.

## 3. Case Presentation

### 3.1. Case 1

A 72-year-old 89-kilogram man with failed back surgery syndrome suffered from low back pain for two years. In addition to the L5/S1 level decompression, the patient also underwent total facetectomy, discectomy, transforaminal lumbar interbody fusion, and internal fixation of the lumbar vertebrae at levels L2/3, L3/4, and L4/5. However, his pain persisted after surgery. He also received an ultrasound-guided caudal injection of triamcinolone, which was ineffective. The patient complained of persistent pain in both buttocks, radiating to the anterior and posterior aspects of the thigh. The numeric rating score (NRS) for pain was 7–8/10 (more severe on the right side). Numbness of the calf and plantar regions was observed bilaterally. 

According to the patient, we decided to perform a bilateral lumbar ESPB at the L3 level for this case, which was the most painful site. Thirty mL of 0.3% ropivacaine was injected under ultrasound guidance, following the above-mentioned injection technique. We followed the patient’s condition every 15 min, but one hour later, there was only a minimal decrease in the NRS to 6–7/10 at rest observed, without any apparent improvement during activity. Two hours after the injection, the patient stated that the pain had reduced by one-third of the pre-treatment intensity (NRS: 4–5/10), both at rest and after activity lasting for 5 min. Three hours after the ESPB injection, the NRS score decreased to half the baseline intensity, even on movement. The effect of pain relief still existed the following morning. After a discussion with the patient, he agreed to be discharged. The patient did not take additional analgesics except his regular medications throughout the admission course. 

### 3.2. Case 2

A 48-year-old 82.5-kilogram man, who had a total hip replacement, was scheduled for wound debridement. The surgical wound was extended to approximately 40 cm, owing to the presence of severe internal infection. The patient received general anesthesia during surgery, and complained of extreme pain at the post-anesthesia care unit. For postoperative pain control, we performed a lumbar ESPB at the L2 level. The pain intensity was examined every 30 min. However, not until 1.5 h after the injection did the patient experience noticeable pain relief, and only mild soreness remained at that time. The patient did not receive other treatment modalities except for a lumbar ESPB before leaving the post-anesthesia care unit. We followed his condition the following morning, and the patient stated that the analgesic effect persisted overnight, and pain control was satisfied with only oral non-opioid analgesics. 

### 3.3. Case 3

We performed a thoracic ESPB on an 82-year-old 58-kilogram man with severe back pain due to failed back surgery syndrome. The patient had undergone multiple spine surgeries at the T7–L4 levels, including vertebroplasty, laminectomy for decompression, and several sessions of interbody fusion with cages at multiple levels, but these were all in vain. The most painful area was at the level of T7–T10 with an NRS of 8/10, and the patient could not stand upright as a result of the pain. On the first day, a bilateral ESPB was administered at the T8 level. Thirty minutes after injection, there was only minimal residual pain at the T7–T10 levels (NRS: 2/10), and no pain was reported at the other sites, both at rest and during activity. The patient was able to stand upright, one hour after the injection. Compared with the baseline, there was also decreased cold sensation noted at the right T11–L3 and left T9–L4 levels. We repeated the ESPB at the right T9 level for recurrent pain (NRS: 7/10) the following day, and the patient experienced similarly swift pain relief. The patient was discharged with an oral analgesic (ultracet), which was consistent with the prescription prior to the procedure.

## 4. Discussion

The analgesic mechanism of ESPB was thought to be the spread of injected local anesthetic along the fascial planes between the erector spinae muscles and the transverse process to dorsal and ventral rami, when it was first published [4]. It was thought to be effective, not only in acute postoperative pain scenarios, but also in chronic pain management [18]. While lumbar ESPBs were widely used in pain control situations, the possible differences in the local anesthetic spread, and the clinical effects between thoracic and lumbar injections were still unclear [17]. There may be anatomical reasons for the difference between thoracic and lumbar ESPBs, mainly the discontinuous lumbar paravertebral space and a more localized lumbar ESPB spread than its thoracic counterpart. The discontinuous lumbar paravertebral space renders lumbar ESPB spread between adjacent levels less reliable than in the thoracic region [19]. Several cadaveric studies attempted to evaluate the actual mechanism of ESPBs in the lumbar region [20,21,22]. Harbell et al. performed nine ESPB injections of 20 mL of methylene blue at L4 on five cadavers, and dissections performed at least two hours after the nine injections found a more localized lumbar ESPB spread than its thoracic counterpart, which they suspected to result from the complex thoracolumbar fascia and thickness of the lumbar musculature [20]. Their study demonstrated a dye spread on dorsal rami mostly between L3 to L5, and no ventral rami were involved. However, the study by Kokar et al. conflicted with these results, suggesting that local anesthetic could still spread through the thickened thoracolumbar fascia [21]. They injected 10 mL of methylene blue at the L4 level, and dissections performed within one hour after the injections found that the dye staining extended from T12 to L5 on dorsal rami, while limited staining on ventral rami was also noted at the single level in half of the specimens. Furthermore, Kokar et al. commented on the study from Harbell et al., with an argument that the spread may remain limited if the needle tip is positioned within the muscles over the transverse process. The above two cadaveric studies with methylene blue may differ from human settings with local anesthetic injection. Similar to conflicting cadaveric results on ventral rami staining in the thoracic ESPB, the discrepancy also exists in lumbar ESPB cadaveric studies. Muscle contraction in the living body induces not only bone movement, but also fascial stretching, with the local anesthetic transported actively via a pump mechanism [23]. Future research should focus on human studies to determine the relationship between the onset time and anterior (or central) contrast spread toward the nervous tissues. 

Besides the conflicting results of anatomical studies about lumbar ESPB injections, the specific difference between thoracic and lumbar ESPBs in clinical effects was not clearly stated. Although many recent publications demonstrated the efficacy of lumbar ESPBs, most of the analyzed outcomes focused on pain scores or opioid consumption [13,14,15,16]. The onset of time was rarely discussed as an issue. Among the three cases in our report, we experienced two patients with chronic low back pain (case 1) and acute postoperative pain (case 2), who received lumbar ESPBs and failed to achieve maximum pain relief until 3 and 1.5 h after injection, respectively. Interestingly, the same local anesthetic formula relieved nearly all pain within 30 min in the third patient with severe chronic back pain who received a thoracic ESPB (case 3). 

In addition to being rare for lumbar ESPB studies to report a specific onset time, the onset gap between thoracic and lumbar injections has never been reported. Forero et al. reported a significant analgesic effect within several minutes of an ESPB at the T5 level with 20 mL of 0.25% bupivacaine [4] (equivalent to a dose of 50 mg bupivacaine), when they first published the ESPB technique. On the other hand, the patients in our study were injected with 30 mL of 0.3% ropivacaine (total dose of 90 mg) in the lumbar region. It is conservatively estimated that the analgesic potency of ropivacaine is approximately 60% of that of bupivacaine [24]. Despite a higher equivalent dose and volume of local anesthetic than previously reported, our lumbar ESPB patients experienced a much slower analgesia onset (Table 1).

Considering the same formula (volume and concentration), our results indicated that ESPBs performed in the lumbar (cases 1 and 2) and thoracic (case 3) regions may have different onset times for the analgesic effect. It seems there is a significant delay in the onset of a lumbar ESPB as opposed to a thoracic ESPB. Even so, case 1 and case 2 still reported significant pain relief in the end. The analgesic effect of lumbar ESPBs may be just as significant as that of thoracic injections after onset. The three cases are summarized in Table 1. 

Owing to a lack of demarcation in these spaces’ boundaries, the local anesthetic spread is usually not confined to the lumbar paravertebral space after injection [17]. Local anesthetic injected at the lumbar region spreads to the psoas muscle and possibly the lumbar plexuses. Several case reports have elaborated on the effects of lumbar ESPBs [6,9,10,11,12,14,15,16,18]; however, the injected local anesthetic formula differed between studies, and some of them combined the formula with lidocaine injections, making the clinical effects not comparable. Whether the local anesthetic regimen affects the onset time of lumbar ESPBs remains uncertain. Based on the possibility of delayed onset of analgesic effect, which was observed in our lumbar ESPB cases using the same formulation of local anesthetic, we argue that if a lumbar ESPB injection is intended for postoperative pain control, the timing of its administration may be of substantial importance in achieving adequate analgesia in time. For instance, if a lumbar ESPB is scheduled for postoperative pain control, the injection could be carried out before the surgery. If not, patients may suffer from a period of post-surgical pain before the onset of the effect.

The other possible solution for the delayed effect of a lumbar ESPB may be adjusting the local anesthetic content, for instance, combining it with a more rapid onset medication such as lidocaine. The optimal local anesthetic concentration and volume for lumbar ESPB injections is another issue under discussion. Tulgar et al. reviewed and summarized reports and studies about lumbar ESPBs in recent years [16]. The local anesthetic formula differed between centers. Most studies used ropivacaine and bupivacaine at 0.375% and 0.25% concentrations, respectively. Some would add 2% lidocaine for a more rapid onset, with epinephrine to avoid local anesthetic systemic toxicity (LAST). While the lumbar ESPB may have a delayed onset of effect, clinicians could consider administering lidocaine to facilitate analgesia. Ahiskalioglu et al. demonstrated a case series of ESPBs at the L4 level as the main anesthetic method for hip fracture surgery in 15 high-risk elderly patients. They administered 20 mL of 0.5% bupivacaine, 10 mL of 2% lidocaine, and 10 mL of normal saline to each candidate [25]. They reported the median onset time of this formula was 30 min, and was adequate to complete the operation when combined with pure intraoperative propofol sedation without other rescue systemic analgesics. While this combination for lumbar ESPBs seemed to have a more rapid onset, Karaca et al. reported a possible LAST case after a lumbar ESPB injection with a similar formula (20 mL of bupivacaine, 10 mL of lidocaine, 9 mL of saline, and 1 mL of methylprednisolone) [26]. The patient lost consciousness and developed a seizure soon after the injection. Moreover, the study by Ahiskalioglu et al. also found the epidural and lumbar plexus spread of local anesthetics, which indicated, in addition to the hemodynamic status, the motor function of lower limbs under a higher volume of lumbar ESPB may be influenced and should be under careful surveillance. In conclusion, the adequate formula and maximum safe dosage of lumbar ESPBs are still not well defined, and caution must be exercised in the case of high-volume ESPB administration.

This report has some limitations. Firstly, this is only a case report; therefore, recall bias unavoidably exists due to its retrospective nature, despite the technique being performed by the same expert with the same formula in all three cases. It would not be easy to draw a firm conclusion at this time with incomplete information about only three cases. We believe the value of this case report is to bring specific problems to attention, and close the gaps of particular issues. Secondly, the basic patient characteristics were not fully matched among the cases, especially case 2, who was much younger with acute postoperative pain, unlike the other two elderly subjects with chronic pain. It is possible that the subjective feeling of pain and drug pharmacokinetics differed between the individuals, especially between those of different ages. However, it is worthwhile to discuss these lumbar ESPB cases using the same formulation of local anesthetic, due to the similar findings regarding delayed onset in different types of patients in different scenarios. Thirdly, the specific cause (such as piriformis syndrome) of the failed back surgery syndrome was not sought before the lumbar ESPB for case 1. Physical examination findings may help create a differential diagnosis, but they are often not reliable in establishing a clear diagnosis in cases of failed back surgery syndrome [2], and ineffective caudal epidural steroid administration may preliminarily rule out the possibility of piriformis syndrome in case 1 [27]. Therefore, due to multiple etiological factors commonly associated with failed back surgery syndrome [2] and the reported effectiveness of bilateral lumbar ESPBs on failed back surgery syndrome [28], we decided to perform a bilateral lumbar ESPB at the most painful level for case 1 without supplementary blocks. It is recommended that a well-designed clinical study be conducted to test more cases of lumbar ESPBs in terms of onset time before extrapolating the current results to other cases.

## 5. Conclusions

Our experience indicates that the time required to attain the peak effect of a lumbar ESPB may be longer than that for a thoracic ESPB. While delayed-onset lumbar ESPB may have some drawbacks for treating immediate postoperative pain, it still could produce significant analgesia upon taking effect when given to patients suffering from hip surgery with large incisions and intractable low back pain. A higher concentration or mixture with a rapid-acting local anesthetic may be necessary to ensure faster action of a lumbar ESPB; however, local anesthetic toxicity and possible motor blockade due to lumbar plexus involvement should be taken into consideration. Additional research is needed to determine whether this type of anesthetic should be administered preoperatively to ensure adequate postoperative analgesia. Therefore, the local anesthetic formula and injection timing should be adjusted for lumbar ESPBs when applied in the perioperative period, in order to make the onset of the analgesic effect coincide with the immediate postoperative pain. Without this concept in mind, clinicians may consider a lumbar ESPB to be ineffective before it takes effect. Future clinical trials should be designed according to our data to compare the lumbar ESPB with its thoracic counterpart regarding onset time.

## Figures and Tables

**Table 1:** Summary of the three cases using the same formulation of local anesthetic.

	Case 1 (Lumbar)	Case 2 (Lumbar)	Case 3 (Thoracic)
Diagnosis	failed back surgery syndrome	avascular necrosis of the right femoral head, severe postoperative pain after debridement	failed back surgery syndrome
Pain intensity before injection	NRS: 7–8/10	Extreme pain	NRS: 8/10
Injection level	L3	L2	T8
Local anesthetic	30 mL of 0.3% ropivacaine (with 1:400,000 epinephrine)		
Time required to achieve maximum pain relief	3 h after injection	1.5 h after injection	30 min after injection

NRS, numeric rating scale.

## Data Availability

Relevant patient data have been presented in the Table.

## Abbreviations

ESPB	Erector spinae plane block
T	Thoracic
L	Lumbar
S	Sacral
NRS	Numeric rating scale

## References

[1] Cohen S.P., Vase L., Hooten W.M. (2021). Chronic pain: An update on burden, best practices, and new advances. Lancet.

[2] Baber Z., Erdek M.A. (2016). Failed back surgery syndrome: Current perspectives. J. Pain Res..

[3] Waszak P.M., Modric M., Paturej A., Malyshev S.M., Przygocka A., Garnier H., Szmuda T. (2016). Spinal Cord Stimulation in Failed Back Surgery Syndrome: Review of Clinical Use, Quality of Life and Cost-Effectiveness. Asian Spine J..

[4] Forero M., Adhikary S.D., Lopez H., Tsui C., Chin K.J. (2016). The Erector Spinae Plane Block: A Novel Analgesic Technique in Thoracic Neuropathic Pain. Reg. Anesth. Pain Med..

[5] Cai Q., Liu G.Q., Huang L.S., Yang Z.-X., Gao M.-L., Jing R., Liu Z., Pan L.-H. (2020). Effects of erector spinae plane block on postoperative pain and side-effects in adult patients underwent surgery: A systematic review and meta-analysis of randomized controlled trials. Int. J. Surg..

[6] Viderman D., Sarria-Santamera A. (2021). Erector spinae plane block in chronic pain management: A scoping review. Tumori.

[7] Melvin J.P., Schrot R.J., Chu G.M., Chin K.J. (2018). Low thoracic erector spinae plane block for perioperative analgesia in lumbosacral spine surgery: A case series. Can. J. Anaesth..

[8] Tulgar S., Senturk O. (2018). Ultrasound guided low thoracic erector spinae plane block for postoperative analgesia in radical retropubic prostatectomy, a new indication. J. Clin. Anesth..

[9] Chung K., Kim E.D. (2018). Continuous erector spinae plane block at the lower lumbar level in a lower extremity complex regional pain syndrome patient. J. Clin. Anesth..

[10] Tulgar S., Senturk O. (2018). Ultrasound guided Erector Spinae Plane block at L-4 transverse process level provides effective postoperative analgesia for total hip arthroplasty. J. Clin. Anesth..

[11] Alici H.A., Ahiskalioglu A., Aydin M.E., Ahiskalioglu E.O., Celik M. (2018). High volume single injection lumbar erector spinae plane block provides effective analgesia for lower extremity herpes zoster. J. Clin. Anesth..

[12] Tulgar S., Ermis M.N., Ozer Z. (2018). Combination of lumbar erector spinae plane block and transmuscular quadratus lumborum block for surgical anaesthesia in hemiarthroplasty for femoral neck fracture. Indian J. Anaesth..

[13] Liu M.J., Zhou X.Y., Yao Y.B., Shen X., Wang R., Shen Q.H. (2021). Postoperative Analgesic Efficacy of Erector Spinae Plane Block in Patients Undergoing Lumbar Spinal Surgery: A Systematic Review and Meta-Analysis. Pain Ther..

[14] Oh S.K., Lim B.G., Won Y.J., Lee D.K., Kim S.S. (2022). Analgesic efficacy of erector spinae plane block in lumbar spine surgery: A systematic review and meta-analysis. J. Clin. Anesth..

[15] Xiao X., Zhu T., Wang L., Zhou H., Zhang Y. (2022). Efficacy of Postoperative Analgesia by Erector Spinal Plane Block after Lumbar Surgery: A Systematic Review and Meta-analysis of Randomized Controlled Trials. Comput. Math. Methods Med..

[16] Tulgar S., Aydin M.E., Ahiskalioglu A., De Cassai A., Gurkan Y. (2020). Anesthetic Techniques: Focus on Lumbar Erector Spinae Plane Block. Local Reg. Anesth..

[17] Kose H.C., Kose S.G., Thomas D.T. (2018). Lumbar versus thoracic erector spinae plane block: Similar nomenclature, different mechanism of action. J. Clin. Anesth..

[18] Durmus I.E., Surucu S., Muz A., Takmaz S.A. (2023). The effectiveness of erector spinae plane block in patients with chronic low back pain. Eur. Rev. Med. Pharmacol. Sci..

[19] Tighe S., Greene M.D., Rajadural N. (2010). Paravertebral block. Contin. Educ. Anaesth. Crit. Care Pain.

[20] Harbell M.W., Seamans D.P., Koyyalamudi V., Kraus M.B., Craner R.C., Langley N.R. (2020). Evaluating the extent of lumbar erector spinae plane block: An anatomical study. Reg. Anesth. Pain Med..

[21] Kokar S., Ertas A., Mercan O., Yildirim F.G., Tastan O.A., Akgun K. (2022). The lumbar erector spinae plane block: A cadaveric study. Turk. J. Med. Sci..

[22] Breidenbach K.A., Wahezi S.E., Kim S.Y., Koushik S.S., Gritsenko K., Shaparin N., Kaye A.D., Viswanath O., Wu H., Kim J.H. (2022). Contrast Spread After Erector Spinae Plane Block at the Fourth Lumbar Vertebrae: A Cadaveric Study. Pain Ther..

[23] Elsharkawy H., Pawa A., Mariano E.R. (2018). Interfascial Plane Blocks: Back to Basics. Reg. Anesth. Pain Med..

[24] Beilin Y., Halpern S. (2010). Focused review: Ropivacaine versus bupivacaine for epidural labor analgesia. Anesth. Analg..

[25] Ahiskalioglu A., Tulgar S., Celik M., Ozer Z., Alici H.A., Aydin M.E. (2020). Lumbar Erector Spinae Plane Block as a Main Anesthetic Method for Hip Surgery in High Risk Elderly Patients: Initial Experience with a Magnetic Resonance Imaging. Eurasian J. Med..

[26] Karaca O., Pinar H.U. (2020). Is high dose lumbar erector spinae plane block safe?. J. Clin. Anesth..

[27] Mullin V., de Rosayro M. (1990). Caudal steroid injection for treatment of piriformis syndrome. Anesth. Analg..

[28] Takahashi H., Suzuki T. (2018). Erector spinae plane block for low back pain in failed back surgery syndrome: A case report. JA Clin. Rep..

